# Protective effect of total saponins of ginseng stems and leaves (GSLS) on chlorpyrifos-induced brain toxicity in mice through the PTEN/PI3K/AKT axis

**DOI:** 10.18632/aging.204374

**Published:** 2022-11-11

**Authors:** Hong Wu, Hongyan Pei, Jinze Liu, Jianning Zeng, Silu Liu, Weijia Chen, Zhongmei He, Rui Du

**Affiliations:** 1 College of Chinese Medicinal Materials, Jilin Agricultural University, Changchun 130118, China

**Keywords:** CPF, HT22 cell line, brain toxic mice, PTEN/PI3K/AKT, apoptosis

## Abstract

Chlorpyrifos (CPF) is a class of toxic compounds which has been widely used in agriculture that can cause multi-organ damage to the liver, kidneys, testes, and nervous system. Currently, most studies on ginseng have concentrated on the roots and rhizomes, and less research has been conducted on the above-ground parts. Our laboratory found that ginseng stem and leaf total saponin (GSLS) features strong antioxidant activity. In this experiment, we selected different concentrations of CPF to induce hippocampal neuronal cell injury model in mice, conducted a cell survival screening test, and also selected appropriate concentrations of CPF to induce brain injury model in mice. CCK-8, flow cytometry, Elisa, Hoechst 33258 staining, Annexin V-FITC/PI staining, HE staining, Morris water maze, and qRT-PCR were adopted for detecting the effects of GSLS treatment on CPF-induced cell viability, mitochondrial membrane potential, reactive oxygen species (ROS) levels, Ca^2+^ concentration and GSLS treatment on CPF-induced brain injury and related signaling in mice, respectively. The effects of GSLS treatment on CPF-induced brain injury and the related signaling pathways in mice were examined. The results showed that GSLS at 60 μg/ml and 125 μg/ml concentrations elevated the viability of CPF-induced HT22 cells, increased mitochondrial membrane potential, depleted ROS, decreased Ca^2+^ concentration, and decreased apoptosis rate. Meanwhile, GSLS treatment significantly reduced CPF-induced escape latency in mice, elevated the number of entries into the plateau and effective area, increased the effective area and target quadrant residence time, as well as improved the pathological damage of mouse hippocampal neurons. The results of mouse brain sections demonstrated that GSLS treatment significantly increased SOD and CAT activities and lowered MDA accumulation in CPF-induced mice. qRT-PCR revealed that PTEN mRNA expression was significantly decreased with PI3K and AKT expression being significantly increased in GSLS-treated CPF-induced mice. Thus, the obtained results indicate that GSLS can effectively antagonize CPF-induced brain toxicity in mice through regulating PTEN/PI3K/AKT pathway.

## INTRODUCTION

Alzheimer’s disease (AD) and Parkinson’s disease (PD) represent two frequently seen chronic neurodegenerative disease, which can influence people globally [1]. In many neurodegenerative diseases, there is a marked increase in neuronal loss and consequent changes in the functional structure of nerve cells compared with controls, whereas the mechanisms driving these neurodegenerative malignancies remain unknown [2, 3]. Recently, as suggested in several articles, organophosphorus (OP) pesticides overuse may become a major factor in neurodegenerative diseases [4–6]. CPF is related to learning and memory dysfunction, increased anxiety, and altered activity and impulsivity [7–9]. Up to the present, related studies have provided sufficient evidence to show that there exists a strong link between CPF exposure, long-term persistent cognitive impairment and increased risk of neurodegenerative diseases [10, 11].

Organophosphorus pesticides (OPs) represent the organic esters that contain phosphorus atoms, among which there are over 150 kinds. Because of the potent capacity to resist insects as well as the extensive applications, OPs have been extensively used worldwide as pesticides against pests and diseases including dichlorvos and chlorpyrifos [12]. Chlorpyrifos (CPF) is considered to be one of the extremely dangerous ones [13]. Chlorpyrifos (CPF) is widely applied in agriculture, especially in rice fields [14]. Nevertheless, as the use of CPF increases, so does the resulting contamination [15]. Because CPF is extensively used in agriculture, its presence can often be detected in vegetables and fruits [16]. CPF and its metabolite chlorpyrifos oxon generate multiple damaging effects on different organs of the body [17]. Due to the lipophilic nature of CPF, the nervous system is the major target of CPF; therefore, it can easily cross and destabilize the blood-brain barrier (BBB), leading to interruption of neurotransmission and neurological dysfunction [18]. As reported in some studies, CPF can induce neurological injuries though at a low dose, including blood-brain barrier (BBB) integrity disruption, dementia, Parkinson’s disease (PD), hyperactivity disorder and, attention deficit [19]. Besides, CPF suppresses the activity of acetylcholinesterase through combining with acetylcholinesterase (AChE) active site, thereby avoiding acetylcholine (ACh) breakdown within the nervous system [20]. Therefore, it leads to ACh deposition within nerve endings and induces persistent cholinergic receptors stimulation, eventually generating paralysis and death [21].

The antioxidant enzymes, such as catalase (CAT), superoxide dismutase (SOD), glutathione reductase (GR) and glutathione peroxidase (GSH-Px), are changed within body after CPF intoxication, and thus it has been proposed that the nerve damage caused by CPF can be undone by anti-oxidative stress [22, 23]. Ginseng is a perennial herb of the genus Ginseng in the family Wujia, and is abundant in northeastern China, Korea, North Korea and eastern Russia, and is known as the “King of All Herbs” [24]. Ginseng has been traditionally used to nourish the vital energy, tranquilize the mind, and educate the mind [25, 26]. Most of the current studies have focused on roots and rhizomes, with less research on aboveground parts. Previous studies in our laboratory revealed that total stem and leaf saponins of ginseng possess strong antioxidant activity. As a result, the present work focused on evaluating how ginseng stem and leaf total saponins protected CPF-induced brain toxicity in mice by assessing their *in vitro* antioxidant activity, anti-cellular oxidative stress, and anti-inflammatory antioxidant activity in mice. [27–29].

## MATERIALS AND METHODS

### Chemicals and reagent kits

CPF (Kaifeng Inspur Chemical Co., Ltd., China) in dimethyl sulfoxide (DMSO, Beijing Solarbio Technology Co., Ltd., China) was adopted to prepare CPF stock solution. Throughout the whole experiment, the <0.05% DMSO dose was maintained. This work obtained normal saline in Heilongjiang Colen Pharmaceutical Co., Ltd. (China) CAT, SOD, MDA and ACh contents were determined with corresponding ELISA kits (Beijing Solarbio Technology Co., Ltd., China). The mouse anti-host primary antibodies were used, with IgG being their isotype. The remaining chemicals were analytically pure bought commercially.

### Plant materials

The total ginsenosides were obtained from Jilin University (Changchun, Jilin, China). Total ginsenoside is dissolved till the specific final concentration prior to utilization.

### Role of CPF treatment in HT22 cell survival rate

CPF’s cytotoxicity to viability of HT22 cells was analyzed by the Cell Counting KIT-8 (CCK-8; Saint Biotech, Shanghai, China). CPF was first diluted with medium for obtaining diverse target doses in the entire experiment. Additionally, HT22 cell density was adjusted with medium to 1 × 10^6^ cells/mL (100 μL/well). Then, CPF (15, 30, 60, 125, 250, 500 μg/ml) at diverse doses was added to treat cells within the 96-well plates for a 3-h period. Afterwards, 100 μL freshly prepared medium that contained CCK-8 solution (10 μL) was added into every well to replace the original medium. Later, HT22 cells were resuspended and incubated for a 30-min period. Absorbance (OD) values in diverse wells were measured with the Cytation5 imaging plate reader (Biotek Instrument, USA) at 450 nm. Afterwards, cell viability following CPF treatments was determined and represented by that of non-treated HT22 cells by using a line chart. Median inhibitory concentrations (IC_50_) together with associated 95% confidence intervals (CIs) were acquired by adopting the logit model.

### Effect of GSLS on HT22 cells treated with CPF

HT22 cells displaying favorable morphological and growth conditions were added into the 96-well plates. When achieving adhesion, non-cytotoxic cells with good cell viability were pre-protected with GSLS (15, 30, 60, 125, 250, 500 μg/ml) for 12 hours. Next, 15 μg/ml CPF was added to treat cells for a 3-h period within the 96-well plates. After that, CCK-8 solution (10 μL) was poured into diverse wells, while OD values were determined with the microplate reader under 37° C after 2 hours at 450 nm.

### Establishment of the AD model

**Figure 1:**
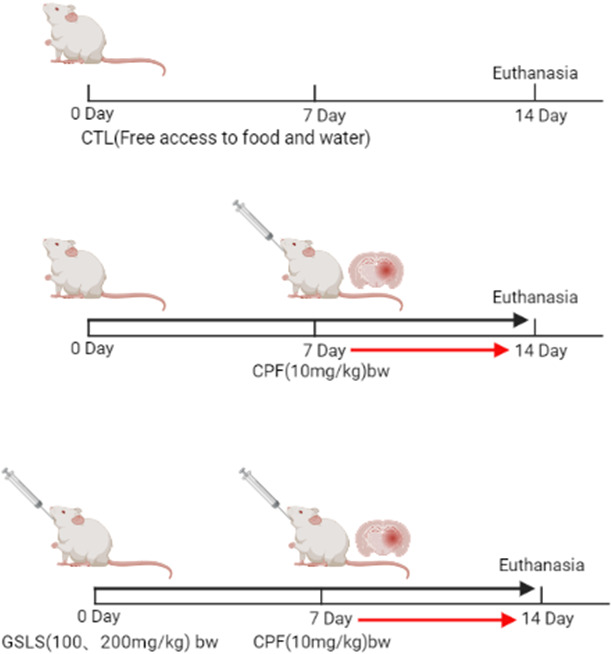
Methods of animal administration in each group (n=10).

This work acquired SPF male Kunming mice weighing 18-22 g from YISI Experimental Animals Co., Ltd. (license No. SCXK (Ji) – 2020 - 0001, Jilin, China). Each experiment was carried out following animal experimental guidelines released by Jilin Agricultural University. Our protocols gained approval from Institutional Animal Care and Use Committee of Jilin Agricultural University with reference to the AVMA Guidelines for Euthanasia of Animals: 2013 Edition (American Veterinary Medical Association) and the 8^th^ edition of Guide for the Care and Use of Laboratory Animals released by the National Academies Press (Washington, D.C.). This study used male rats because chronic research finds that males show high sensitivity and susceptibility to chemical-triggered toxicity effect compared with females [30]. Mice were divided as 4 groups, respectively, control, CPF, CPF (10mg/kg) + GSLS (100mg/kg) and CPF (10mg/kg) + GSLS (200mg/kg). There are 10 mice in each group. The control mice were administered with saline at an equivalent amount for two weeks. Mice in model group were administered with saline at an equivalent amount for the first week and CPF (10mg/kg/d) in the second week. The low- and high-dose GSLS groups were pre-protected at 100 and 200 mg/kg/d for one week, in combination with CPF (10mg/kg/d) + GSLS (100mg/kg/d) and CPF (10mg/kg/d) + GSLS (200mg/kg/d) for the second week respectively. All modes of administration were intragastric. The dosage regimen is presented in Figure 1.

### Hoechst 33342 staining

Hoechst 33342 is a kind of blue fluorescent dye used to stain DNA [31]. In this assay, a normal clean coverslip was taken, soaked in 70% ethanol for 5 min, blow-dried in a sterile ultra-clean table, and washed thrice with PBS solution, and then with cell culture solution. Later, the coverslip was placed in a six-well plate and the cells were grown overnight, so that the cells reached about 50%-80% confluency. After stimulating the cells to apoptosis, the culture medium was aspirated, 0.5 ml fixative solution (4% paraformaldehyde) was added to fix for 10 min. Then, the fixative was removed and rinsed by PBS for 3 min twice to aspirate liquid under manual shaking several times. Then, cells were stained with Hoechst 33258 solution (0.5 ml, 5 mg/L) for a 5-min period under manual shaking several times. After washing twice with PBS for 3 min each, anti-fluorescence quenching blocking solution (one drop) was placed on the slide and covered with a coverslip with cells attached to it, so as to avoid air bubbles as much as possible. Finally, cells were dried at room temperature and observed with the fluorescent microscope, followed by photographing.

### Annexin V-FITC/PI staining

Annexin V-FITC/PI staining can be applied in detecting early apoptosis and distinguish it from necrosis or late apoptosis [32]. After digestion, this work harvested HT22 cells into the 10 ml centrifuge tube with 1×10^6^/mL of cells per sample. Then, the cells were subject to 5-min centrifugation at 500~1000r/min, followed by elimination of medium. After washing by an incubation buffer once, cells were subject to 5-min centrifugation at 500~1000r/min. The cells were resuspended with 100ul of labeling solution. Meanwhile, this work added Annexin V-FITC (5μl) as well as PI (5μl) solution. After adding fluorescent (SA-FLOUS) solution, cells were subject to 20-min incubation in dark under 4° C and shaking from time to time. The results were detected by flow cytometry.

### Reactive oxygen species (ROS) measurement

ROS contents were determined by fluorescent staining imaging. HT22 cells (1×10^5^/well) were inoculated in the 6-well culture plates, followed by 8-h culture to the stably adherent status. Thereafter, CPF and CPF+GSLS were added to treat cells for a 24-h period, respectively, in line with specific protocols. The ROS probe 2,7-dichlorofluorescein diacetate (DCFH-DA) was later added to treat cells for a 30-min period, followed by rinsing by PBS (2.89 g/L Na_2_HPO_4_, 0.8 g/L NaCl, 0.2 g/L KCl, 0.2 g/L KH_2_PO_4,_ pH 7.4) twice and finally ROS contents were determined through fluorescence microscopy (Thermo Fisher Scientific, USA).

### Ca^2+^content in cells

Ca^2+^ contents in cells were determined by Cal-630”AM (Beijing Baiaolaibo Technology Co., Ltd., China). 2 mL AD cells (1×10^5^/ well) was added at the bottom of 6-well plates overnight. On the day of testing, medium (conditioned medium) in plates incubated overnight with cells was removed and stored at 37° C, 5% CO_2_. Cal-630”AM Diluent, the sample was diluted with medium at 1:1000 to a final concentration of 2 μM. Later, all wells were introduced with 2 μM in DMSO, followed by incubation of dye mixture in an incubator (37° C, 5% CO_2_) for 60 min. Flow cytometry (FCM) was conducted to measure fluorescence intensity.

### Cellular mitochondrial membrane potential (MMP) measured by FCM

After inoculation into the 6-well plate at 1×10^5^/well, cells were subject to resuspension within the medium as well as later 20-min incubation using JC-1 staining solution under 37° C. Immediately thereafter, plate was rinsed by JC-1 dye twice. At last, cells were resuspended with staining dye (500 μL), followed by analysis with FCM.

### Morris water maze (MWM) test

MWM (Chengdu TME Technology Co., Ltd., Chengdu, China) was adopted for evaluating the mouse learning and memory ability according to previous description. It includes one circular pool and an automatic video recording and analysis system. First, titanium dioxide was put into the pool to make it milky white, and the platform was placed at 2 cm underwater. The head of each animal was dyed yellow, and put into the pool wall at 1/2 radian of the quadrant. Mice were guided towards the platform and trained twice a day if they could not find the platform within 120 s. The positioning navigation experiment was conducted on 1-4 days, while space exploration on the 5th day. All data were combined as the learning and memory achievements.

### HE staining

Histopathological examination was carried out according to previous description [33]. The dissected brain tissues were subject to overnight fixation with 4% formaldehyde under 4° C, paraffin-embedding, and slicing into the 5-pm sections when they were dehydrated and permeabilized. After xylene deparaffinage, sections were subject to gradient ethanol rehydration and HE staining. Hippocampal tissues were examined for histopathological alterations with the optical microscope (Olympus Optical Co Ltd; Tokyo, Japan) under the magnification of 400×.

### Measurement of SOD, MDA and CAT contents within hippocampal samples

This work conducted SOD assay according to SOD activity for inhibiting the reduction of nitroblue tetrazolium [34]. This work determined CAT activity according to description of Weydert [35]. Malondialdehyde (MDA) was measured based on the method of this study [36]. After animal sacrifice, this work harvested brain tissues, which were processed by homogenization and 10-min centrifugation at 4000r/min to analyze biochemical parameters. Later, SOD, MDA and CAT activities were measured by relevant kits (Beyotime, Shanghai, China) in line with specific protocols.

### Quantitative real-time PCR (qRT-PCR)

Pro-inflammatory central cytokines were determined following the administration of CPF, GSLS and saline gavage at the corresponding time point (24 h after the last gavage) at which various behavioral indicators were collected. qRT-PCR, the sensitive approach to detect the low central cytokine levels, was applied in the present work for assessing PTEN, PI3K and AKT expression within the mouse brain. By adopting the 7500 real-time PCR thermal cycling system, this work adopted TaqMan™ probe to measure RNA content with related primers. Brain tissues dissected in one rat were operated thrice of every gene, while relative amounts in raw fluorescence intensity were compared among different treatments. Table 1 displays target mRNA primers utilized in RT-PCR (Table 1), with ß-actin being the housekeeping gene.

**Table 1:** Gene-specific primers used in qRT-PCR.

**Gene**	**Forward sequence (5’ to 3’)**	**Reverse sequence (3’ to 5’)**
PTEN	TCCCAGTCAGAGGCGCTATGTA	CCTTTAGCTGGCAGACCACAAAC
PI3K	TGTGGCACAGACTTGGTGTT	TTCTTCCCTTGAGATGTCTCCC
AKT	CCGCCTGATCAAGTTCTCCT	TTCAGATGATCCATGCGGGG
β-actin	ATTGTCCACCGCAAATGCTTC	AAATAAAGCCATGCCAATCTCGTC

### Statistical processing

Statistical processing was performed using GraphPad Prism 8. The significance of difference of diverse groups was analyzed through adopting Tukey’s test and one-way ANOVA. Results were represented by mean±SD. Diverse superscript letters indicated statistical significance (*P<0.05*).

## RESULTS

### Role of CPF treatment in HT22 cell viability

**Figure 2:**
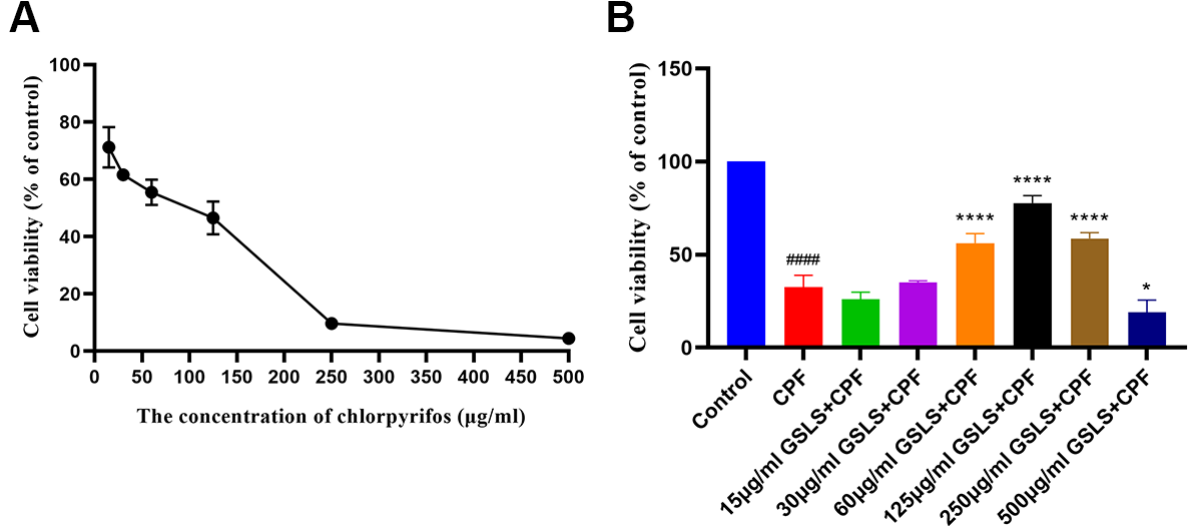
(**A**) The effect of CPF on viability of HT22 cells. (**B**) The effect of GSLS treatment on HT22 cells induced by CPF. ^#^p < 0.05, ^##^p < 0.01, ^###^p < 0.001, ^####^p < 0.0001 vs. control group; ^*^p < 0.05, ^**^p < 0.01, ^***^p < 0.001, ^****^p < 0.0001 vs. CPF group.

CPF has been suggested with serious toxicity to animals. As a result, CCK-8 assay was conducted to analyze the role of CPF treatment in viability of HT22 cells. According to Figure 2A, CPF at diverse doses suppressed HT22 cell survival. The reduction of decreased viability might be associated with CPF treatment dose-dependently. IC_50_ of CPF within HT22 cells was 58.29 μg/ml at 3h. According to our analysis results, at the CPF of 15 μg/ml, CPF-treated HT22 cell viability showed significant difference compared with control (*p<0.05*), consequently this work chosen CPF (15 μg/ml) to be the cell-modeling dose.

### GSLS improves the spatial learning and memory defects in AD mice induced by CPF

CCK-8 assay was performed to explore GSLS’ mitigating effect on the CPF-induced decrease in HT22 cell viability (Figure 2B). 15μg/ml CPF dramatically reduced cell viability (*P<*0.05). In the subsequent experiments, the optimal concentration of GSLS found by the stimulation performed was 125 μg/ml, corresponding to over 80% viability of HT22 cells.

### Analysis of Hoechst 33258 and Annexin V/PI staining results

**Figure 3:**
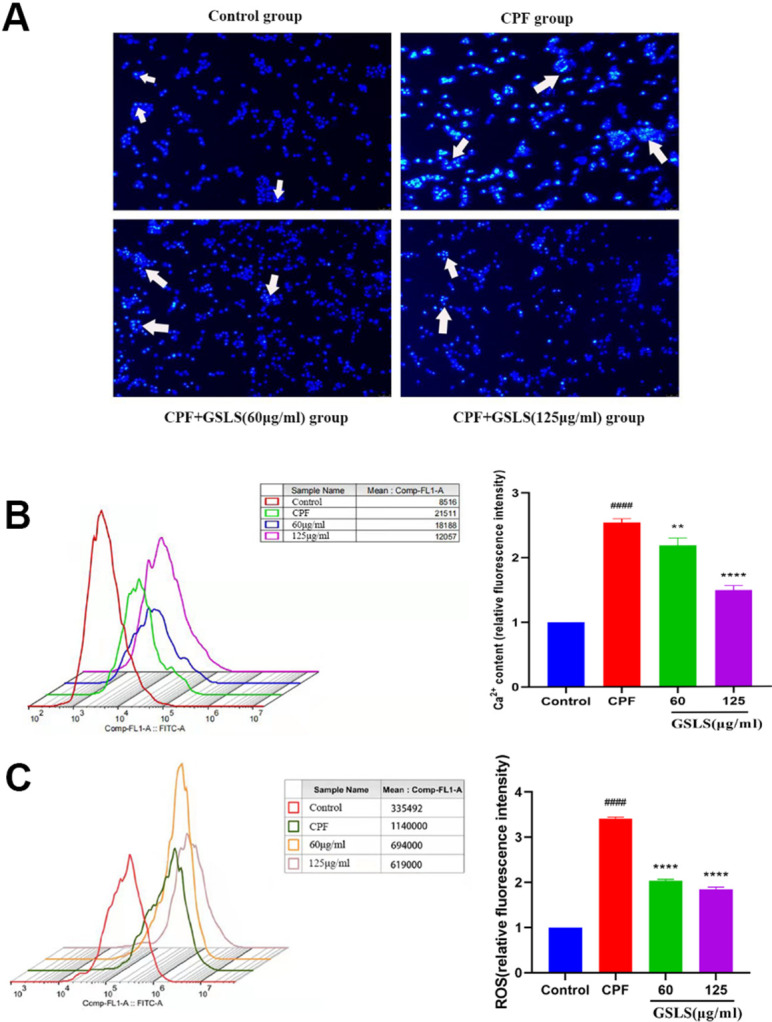
(**A**) Hoechst 33342 staining assessment. (**B**) Detection of intracellular Ca^2+^ concentration. (**C**) Detection of reactive oxygen species (ROS). ^#^p < 0.05, ^##^p < 0.01, ^###^p < 0.001, ^####^p < 0.0001 vs. control group; ^*^p < 0.05, ^**^p < 0.01, ^***^p < 0.001, ^****^p < 0.0001 vs. CPF group.

**Figure 4:**
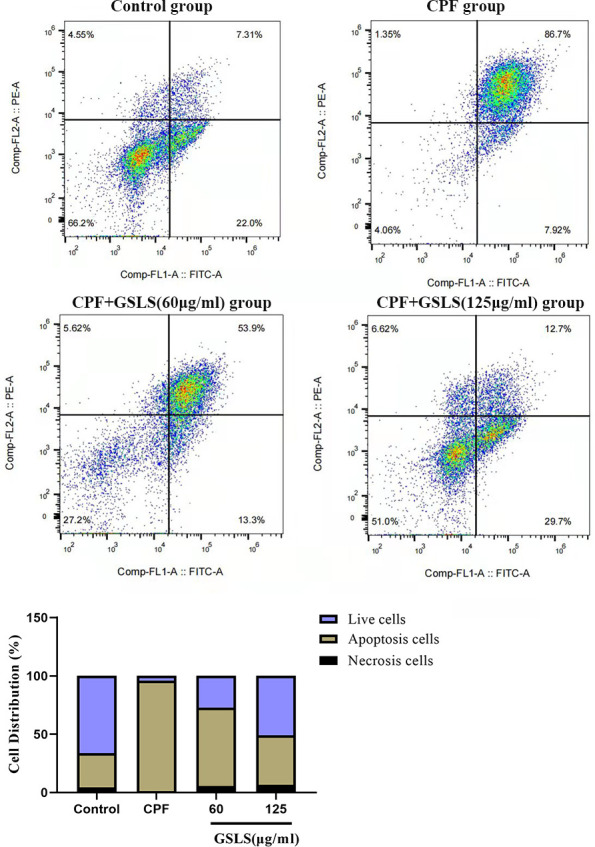
**Annexin V/PI staining results.** Flow cytometry was detected the proportion of apoptotic cells after CPF exposure.

Apoptosis usually accompanies with nuclear morphological alterations, including nuclear condensation, chromosomal margin lobulation and chromatin granulation. Nuclear morphological alterations were monitored by Hoechst 33258 staining. As shown in Figure 3A, for model group, nuclei exhibited the increased fluorescence intensity compared to control group. The chromatin was concentrated and the nuclei became smaller. Relative to model group, GSLS exposure remarkably declined nuclear fluorescence intensity, with insignificant nuclear apoptotic features, conforming to the control group. As shown in Annexin V/PI analysis (Figure 4), model group had apparently more apoptotic cells (*P<*0.05) and dramatically decreased viable cells (*P<*0.05) relative to control. Relative to model group, apoptotic cells were significantly reduced (*P<*0.05), while viable cells dramatically elevated (*P<*0.05) after GSLS treatment. Consequently, CPF promoted HT22 cell apoptosis, while GSLS protected against CPF-mediated HT22 cell apoptosis.

### GSLS attenuated CPF-mediated oxidative stress (OS)

For evaluating how GSLS prevented the impact of CPF treatment on HT22 cell OS, the ROS level was detected. The generation of ROS in HT22 cells was detected using fluorescence microscope (Figure 3C). DCFH-DA probe is able to penetrate the cell membrane, finally hydrolyzed for forming DCFH. ROS contents in cells oxidize DCFH into fluorescent DCF. Therefore, fluorescence intensity represents ROS content, and the experimental results are analyzed by Image J software. Relative to control, CPF markedly elevated ROS production (*P<*0.05). Following GSLS exposure, ROS level decreased significantly, and fluorescence intensity dramatically decreased compared with CPF group (*P<*0.05).

### Effect of GSLS treatment on Ca^2+^ concentration in HT22 cells exposed to CPF

To evaluate effect of GSLS treatment on Ca^2+^ homeostasis in HT22 cells exposed to CPF, the Ca^2+^ concentration level was measured (Figure 3B). We detected that whether GSLS could reduce Ca^2+^ overload in HT22 cells exposed to CPF by flow cytometry. CPF exposure markedly reduced peak value of intracellular Ca^2+^ relative to control (*P<*0.05). Compared with CPF group, intracellular Ca^2+^ concentration elevated markedly following GSLS exposure (*P<*0.05).

### GSLS alleviates CPF-mediated cell apoptosis

**Figure 5:**
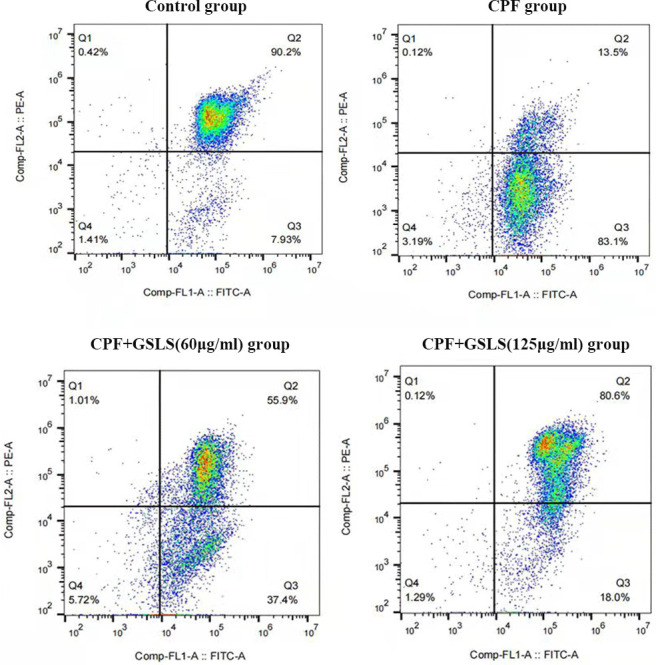
**Detection results of mitochondrial potential (ΔΨm).** The ΔΨm in each group stained with JC-1 and calculated the fluorescence by flow cytometry.

For illustrating how CPF induced apoptosis, MMP was analyzed. JC-1 is the indicator for ΔΨm, which was adopted in the present work for analyzing mitochondrial depolarization. According to Figure 5, FCM demonstrated the role of CPF treatment in markedly reducing red/green fluorescence ratio, demonstrating the decreased ΔΨm as well as mitochondrial depolarization, finally promoting apoptosis. Nevertheless, compared with GSLS treatment group, red/green fluorescence ratio markedly returned to normal level after GSLS treatment. GSLS saved the transformation of ΔΨm, which indicated that GSLS reduced mitochondrial dysfunction, thus inhibiting CPF-induced apoptosis.

### GSLS enhanced CPF-mediated mouse learning and memory abilities.

**Figure 6:**
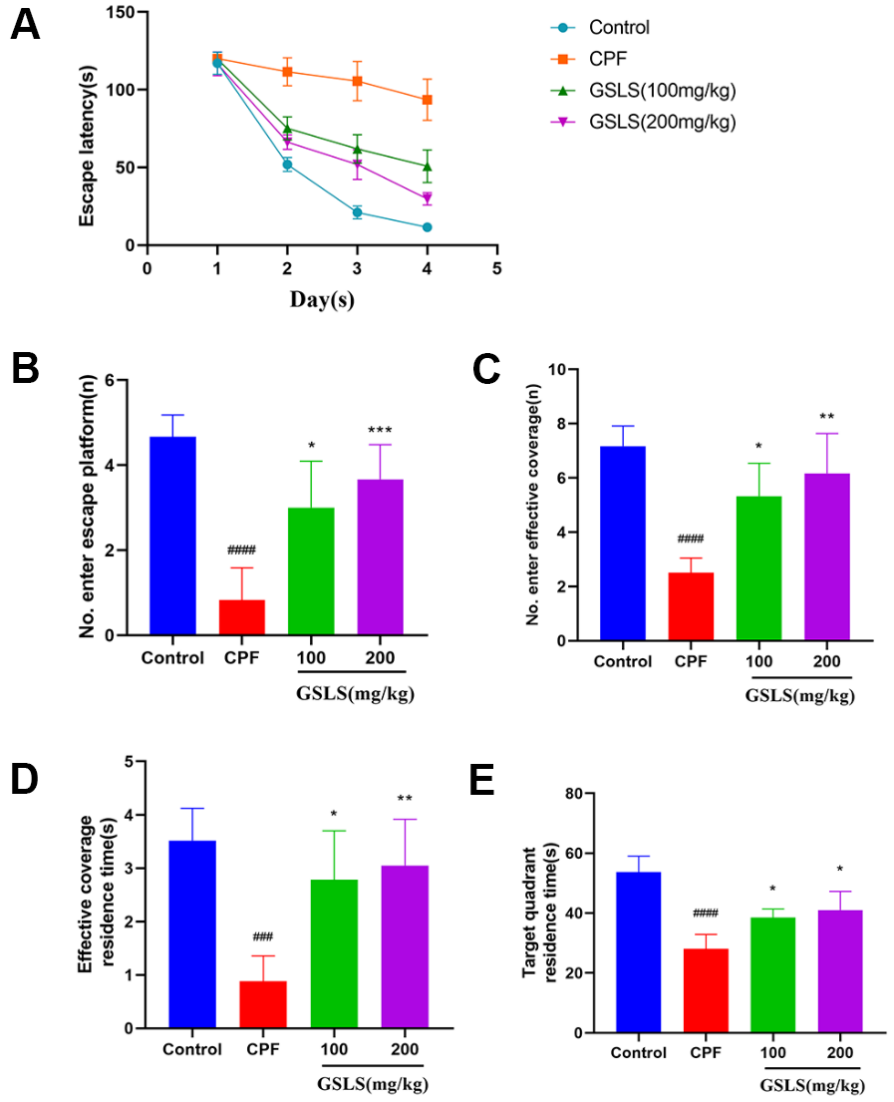
**The experimental results of Morris water maze.** (**A**) The escape latency. (**B**) Number of entries onto the escape platform. (**C**) Number of entries into the effective coverage area. (**D**) Effective coverage area residence time. (**E**) Target quadrant residence time. ^#^p < 0.05, ^##^p < 0.01, ^###^p < 0.001, ^####^p < 0.0001 vs. control group; ^*^p < 0.05, ^**^p < 0.01, ^***^p < 0.001, ^****^p < 0.0001 vs. CPF group.

**Figure 7:**
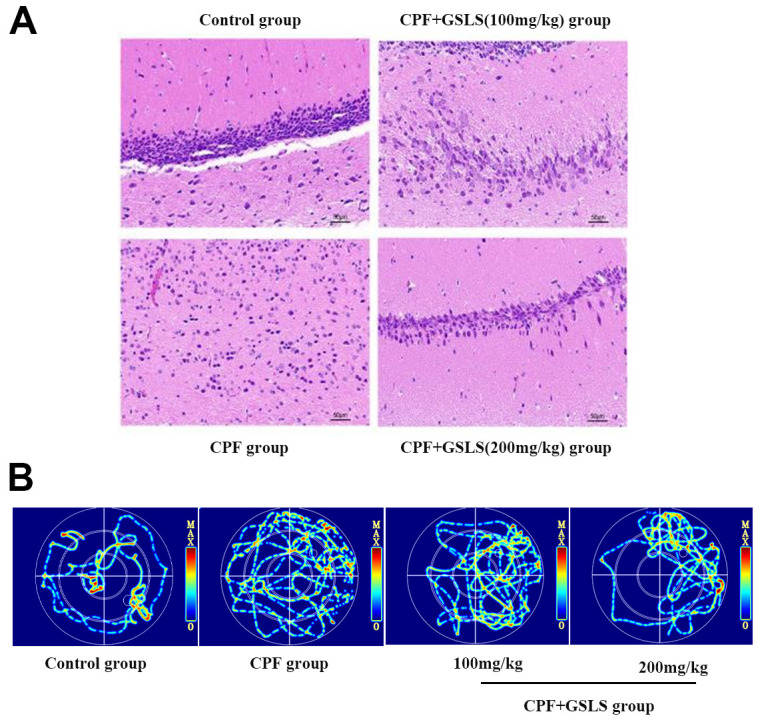
(**A**) HE staining of pathological changes in mouse hippocampi (magnification: 400 ×, n = 10 in each group). (**B**) Thermal infrared track diagram of mouse moving in Morris water maze.

MWM test assessed spatial learning and memory abilities through ELT and swimming thermal infrared trajectory. The navigation results (Figure 6A) demonstrated that relative to control group, CPF group had apparently longer mouse escape latency (*p<*0.05). Relative to model mice, GSLS mice had markedly reduced mouse escape latency (*p<*0.05). As revealed by the results of space exploration (Figure 6B–6E), relative to control group, times of mice climbing platform, entering the effective area, the time of staying in the effective area and in the target quadrant remarkably decreased among CPF mice (*p<*0.05). Compared with model mice, these above four mouse indexes of GSLS group dramatically improved. On day 5 of space exploration experiment, the thermal infrared track of mice in each group (Figure 7B) showed that normal mice directly swam within target quadrant and found that hidden underwater platform. Nonetheless, CPF mice swam aimlessly along pool edge. Following GSLS exposure, those treated mice found hidden platform within target quadrant. Of those four treatments, GSLS high-dose mice had the optimal performance.

### HE staining analysis

Control mice had larger conical hippocampal neurons, neatly arranged, with well-defined cytoplasm and nuclei, whereas those in the CPF group were significantly reduced, with loosely arranged disorganized cells and markedly atrophied cytoplasmic lysates. The histopathological results showed that CPF injured hippocampal neurons. GSLS exposure improved pathological injury, with better improvement in high-dose group (Figure 7A).

### Effects of GSLS on CAT, MDA and SOD within mouse brains induced by CPF

**Figure 8:**
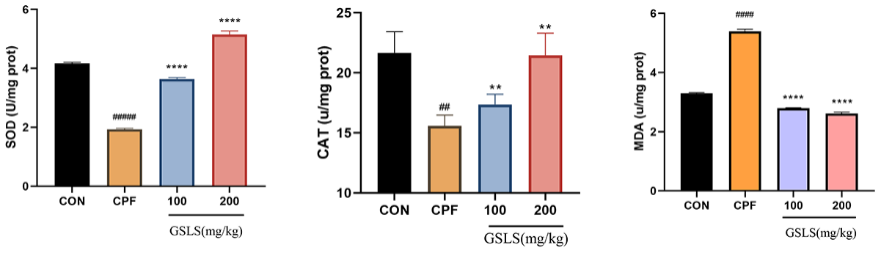
**The effect of CPF and/or GSLS on the antioxidant function.** The contents of assay kits of SOD, CAT and MDA have expressed as the means ± standard deviations (n = 3). ^#^p < 0.05, ^##^p < 0.01, ^###^p < 0.001, ^####^p < 0.0001 vs. control group; ^*^p < 0.05, ^**^p < 0.01, ^***^p < 0.001, ^****^p < 0.0001 vs. CPF group.

In Figure 8, relative to control mice, control group showed markedly lower CAT, SOD activities (*P<0.05*). By contrast, compared with control group, MDA content of model group increased significantly. Relative to model group, GSLS group had markedly increased CAT, SOD activities of GSLS group, while MDA content was significantly decreased. It is suggested that GSLS can promoted CAT and SOD activities while reducing MDA content of mice induced by CPF.

### Effects of GSLS treatment on PTEN/PI3K/AKT pathway related gene expression in CPF exposed mice

**Figure 9:**
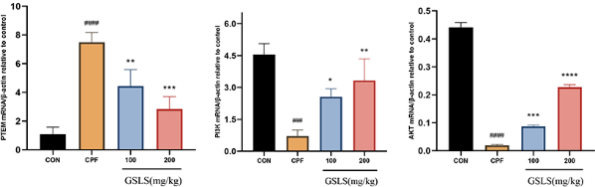
**The mRNA expression levels of indicators in the mitochondrial apoptosis and PTEN/PI3K/AKT pathway under CPF and/or GSLS treatments in HT22 cells.** Values are expressed as the means ± standard deviations (n = 3). ^#^p < 0.05, ^##^p < 0.01, ^###^p < 0.001, ^####^p < 0.0001 vs. control group; ^*^p < 0.05, ^**^p < 0.01, ^***^p < 0.001, ^****^p < 0.0001 vs. CPF group.

After a week of pre-protection with GSLS, mice were exposed to CPF for another week. The PTEN/PI3K/AKT pathway-associated gene levels in mouse brain was detected by qRT-PCR (Figure 9). Relative to control mice, PTEN mRNA expression markedly increased (*p<*0.05). By contrast, AKT and PI3K mRNA and protein expression declined with statistical significance (*p<*0.05). Therefore, PTEN showed negative effect on regulating PI3K/AKT pathway. Additionally, PI3K/AKT expression is positively related to GSLS content, indicating that CPF suppressed PI3K/AKT pathway via the activation of PTEN effect (*p<*0.05), while GSLS activates PI3K/AKT pathway by inhibiting PTEN effect.

## DISCUSSION

Organophosphorus (OP) compounds have been under the continuous use in agricultural pest control worldwide for 50 years, which can be ascribed to their wide range of uses as insecticides, helminthicides, nematicides, fungicides, and herbicides, typically, OP compounds alone account for 38% of the total pesticides used globally [37]. CPF is the common organophosphate (OP) insecticide extensively utilized as an insecticide in agriculture. Nevertheless, its use indoors and outdoors can cause acute lethal effects and side effects on animals and humans [38, 39]. The present work focused on investigating the neurotoxicity of CPF as well as neuroprotection of GSLS in mice. According to our results, CPF interfered with cholinergic transmission through inhibiting AChE [40]. Acetylcholinesterase is found mainly in the neuromuscular junction, plasma, liver, and erythrocytes, and AChE specific catalytic activity reduces ACh signaling and determines a person’s cholinergic state. Acetylcholinesterase exerts an important effect on acetylcholine metabolism, while the dysregulated AChE generates diverse pathological states [41]. Inhibition of AChE leads to the accumulation of acetylcholine in the synapses, which leads to continuous overstimulation of the nervous system, thus probably leading to the death of the organism [42]. Different doses of CPF have been reported to inhibit acetylcholinesterase, causing acetylcholine accumulation in synaptosomes, thereby inducing learning and memory dysfunction [43, 44]. Evidently, CPF can inhibit acetylcholinesterase activity through promoting serine site phosphorylation [45].

The brain biochemical integrity has a critical role in the proper central nervous system (CNS) functioning. OS is a factor causing biochemical damage in the brain, which takes place when there is an overproduction of free radicals due to an inadequate ability to counteract antioxidation. Brain consumes the highest oxygen and shows the highest lipid contents, shows a high susceptibility to OS [46]. Exposure to toxic elements in the environment can cause neuronal damage and cognitive deficits [47]. MDA represents a lipid peroxidation by-product, which is adopted to be the OS marker [48]. In this study, redox homeostasis was disturbed in mice after chronic CPF treatment, as evidenced by the increased MDA contents.

Enzymatic activity alterations are the early biochemical sign of CPF toxicity [49]. ROS are detrimental to human health, while CAT and SOD represent two main antioxidant enzymes resisting ROS [50]. When CPF accumulates in the body, the scavenging ability of intracellular antioxidant enzymes is disrupted, which subsequently causes oxidative stress as well as induces neurotoxicity in the brain, resulting in brain damage [51–53]. In the present study, the SOD and CAT contents in the brains of CPF mice decreased, indicating that the superoxide anion was not eliminated and that CPF damaged the nervous system of mice through ROS.

ROS induces apoptosis through multiple pathways, besides, excessive ROS accumulation leads to mitochondrial damage, which is mainly characterized by a decrease in mitochondrial membrane potential and cellular calcium overload, leading to apoptosis [54–56]. In this study, Annexin V/PI double-staining method was utilized to investigate CPF’s function in HT22 cell apoptosis. To further elucidate the apoptotic mechanism, the mitochondrial membrane potential (MMP) of HT22 cells exposed to CPF was detected by JC-1, and the mitochondrial membrane potential of HT22 cells exposed to CPF was found to be decreased significantly. This is consistent with our speculation that CPF-induced apoptosis in neuronal cells may occur in the mitochondrial pathway.

Ginsenosides Rg1, Rb1, Rb2, Rb3 and Rd are present in the stem and leaves of ginseng as non-medicinal parts of ginseng, which are similar to ginseng roots and rhizomes, and have strong pharmacological activities and bio-exploitation value, as confirmed in previous studies [57, 58]. It has been pointed out that total ginseng stem and leaf saponins have strong anti-myocardial ischemic, neuroprotective, anti-oxidative stress, anti-aging and other pharmacological activities [59, 60]. As suggested by Makeen et al., CPF-mediated antioxidant enzyme inactivation in mouse hippocampal neuronal cells led to oxidative stress in the organism and increased ROS *in vivo* [61]. Our study showed that GSLS significantly attenuated CPF-induced oxidative stress in mouse hippocampal neuronal cell lines. GSLS administration remarkably increased SOD and CAT levels, decreased MDA content, and inhibited ROS levels, thereby attenuating CPF-mediated OS.

GSLS is recently suggested to mitigate cellular injury resulting from a variety of factors [62]. PTEN represents the key factor regulating PI3K/AKT signaling, and the PTEN/PI3K/AKT pathway can modulate cellular growth, which also exerts an important effect on cell injury [63, 64]. It has been reported that ginsenosides can down-regulate PTEN level, thereby causing the PTEN/PI3K/AKT pathway activation and decreasing hydrogen peroxide-induced apoptosis [65]. Notably, the overproduction of ROS induces a rise in PTEN expression, which inhibits PI3K/AKT activation and ultimately leads to apoptosis [66]. Additionally, the researchers found that OS modulated PTEN/PI3K/AKT pathway and promoted cellular toxicological processes [67]. Decreased PTEN/PI3K/AKT pathway activation is observed after induction of ROS release from cells [68]. According to our staining and flow cytometry results in this work, CPF triggered excessive ROS production in mouse neuronal cells, promoting OS as well as cell apoptosis, but GSLS treatment significantly attenuated the CPF-induced cell apoptosis. Based on JC-1 analysis, GSLS abolished CPF-induced MMP loss. Moreover, according to qRT-PCR results, PTEN expression increased while PI3K/AKT level was inhibited in CPF-induced mouse brain tissues, thus promoting apoptosis and necrosis of neuronal cells, and GSLS treatment antagonized this process and thereby attenuated cell apoptosis. To sum up, our research suggests that GSLS may prevent the process of CPF-mediated cell apoptosis through down-regulating PTEN while promoting PI3K/AKT pathway, which can also reflect the neuroprotective effect of GSLS.

## CONCLUSIONS

In summary, it is demonstrated in this work that CPF exposure can trigger apoptosis in mouse hippocampal neurons by blocking the antioxidant enzyme activity, which will cause ROS, trigger OS, and enhance mitochondrial apoptotic pathway. Besides, GSLS abolishes the above impacts by PTEN/PI3K/AKT pathway. Moreover, our results provide a certain foundation for future GSLS treatment of pesticide-induced brain toxicity injury and promotion of animal health.
