# Correction for: Protective effect of total saponins of ginseng stems and leaves (GSLS) on chlorpyrifos-induced brain toxicity in mice through the PTEN/PI3K/AKT axis

**DOI:** 10.18632/aging.204951

**Published:** 2023-08-14

**Authors:** Hong Wu, Hongyan Pei, Jinze Liu, Jianning Zeng, Silu Liu, Weijia Chen, Zhongmei He, Rui Du

**Affiliations:** 1 College of Chinese Medicinal Materials, Jilin Agricultural University, Changchun 130118, China

**Keywords:** CPF, HT22 cell line, brain toxic mice, PTEN/PI3K/AKT, apoptosis

**This article has been corrected:** The authors found an error in **Figure 3A:** the magnification used for the images of Hoechst 33342-stained cells shown is not uniform. The authors corrected the error with representative images from the original experiments. The presented correction does not affect the results or conclusions of this article.

Corrected **Figure 3** is presented below.

**Figure 3:**
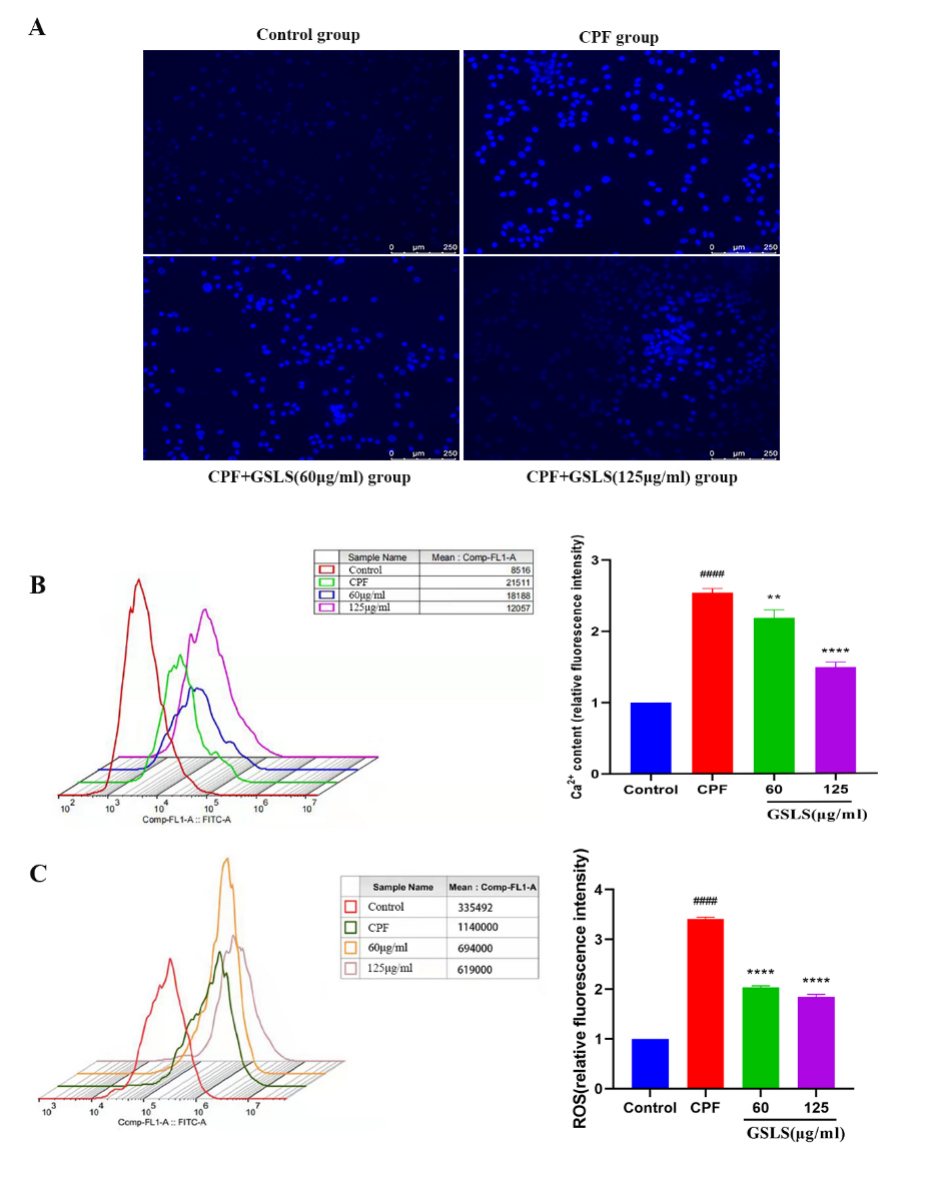
(**A**) Hoechst 33342 staining assessment. (**B**) Detection of intracellular Ca^2+^ concentration. (**C**) Detection of reactive oxygen species (ROS). ^#^p < 0.05, ^##^p < 0.01, ^###^p < 0.001, ^####^p < 0.0001 vs. control group; *p < 0.05, **p < 0.01, ***p < 0.001, ****p < 0.0001 vs. CPF group.

